# An Innovative Lipidomic Workflow to Investigate the Lipid Profile in a Cystic Fibrosis Cell Line

**DOI:** 10.3390/cells9051197

**Published:** 2020-05-12

**Authors:** Michele Dei Cas, Aida Zulueta, Alessandra Mingione, Anna Caretti, Riccardo Ghidoni, Paola Signorelli, Rita Paroni

**Affiliations:** 1 Laboratory of Clinical Biochemistry and Mass Spectrometry, Department of Health Sciences, Università degli Studi di Milano, 20142 Milan, Italy; rita.paroni@unimi.it; 2 Laboratory of Biochemistry and Molecular Biology, Department of Health Sciences, Università degli Studi di Milano, 20142 Milan, Italy; aidazulueta@gmail.com (A.Z.); alessandra.mingione@unimi.it (A.M.); anna.caretti@unimi.it (A.C.); riccardo.ghidoni@unimi.it (R.G.); paola.signorelli@unimi.it (P.S.); 3 Aldo Ravelli Center for Neurotechnology and Experimental Brain Therapeutics, Department of Health Sciences, Università degli Studi di Milano, 20142 Milan, Italy

**Keywords:** lipidomics, OMICS, untargeted analysis, cystic fibrosis, biomarker, sphingolipid, membrane composition, cell structure

## Abstract

Altered lipid metabolism has been associated to cystic fibrosis disease, which is characterized by chronic lung inflammation and various organs dysfunction. Here, we present the validation of an untargeted lipidomics approach based on high-resolution mass spectrometry aimed at identifying those lipid species that unequivocally sign CF pathophysiology. Of n.13375 mass spectra recorded on cystic fibrosis bronchial epithelial airways epithelial cells IB3, n.7787 presented the MS/MS data, and, after software and manual validation, the final number of annotated lipids was restricted to n.1159. On these lipids, univariate and multivariate statistical approaches were employed in order to select relevant lipids for cellular phenotype discrimination between cystic fibrosis and HBE healthy cells. In cystic fibrosis IB3 cells, a pervasive alteration in the lipid metabolism revealed changes in the classes of ether-linked phospholipids, cholesterol esters, and glycosylated sphingolipids. Through functions association, it was evidenced that lipids variation involves the moiety implicated in membrane composition, endoplasmic reticulum, mitochondria compartments, and chemical and biophysical lipids properties. This study provides a new perspective in understanding the pathogenesis of cystic fibrosis and strengthens the need to use a validated mass spectrometry-based lipidomics approach for the discovery of potential biomarkers and perturbed metabolism.

## 1. Introduction

Lipids are a fundamental component of cellular membranes and signaling molecules regulating cellular functions that include energy storage, cell proliferation and death, stress response, and inflammation. Alterations in lipids metabolism are associated and suggested as causative for the pathophysiology of inflammation-related diseases such as neurodegenerative diseases (i.e., Alzheimer’s and Parkinson’s), diabetes, obesity, atherosclerosis and cardiovascular diseases, non-alcoholic fatty liver disease, cancer, obstructive sleep apnea, and respiratory diseases [1,2]. Thus, lipids are not only modulated by diseases but also recognized as therapeutic targets. Lipidomics is the most powerful tool to approach the study of lipids-related diseases. The increasing popularity of the lipidomics approach is strictly connected to the progress in the related analytical techniques, especially mass spectrometry. This large-scale technique can cover the whole human lipidome, comprising from 10 to 100-thousand different chemical entities in a complex biological system. Lipids can be studied by two approaches: targeted or untargeted lipidomics. Targeted methods are a high-sensitive analysis dedicated to the identification and quantification of known classes of lipids, whereas nontargeted methods, usually employing high-resolution mass spectrometry, aim to identify and semi-quantify every likely lipid species contained in the samples [3,4,5]. Employing this technique, different tasks can be performed: (1) characterization, identification, and quantification of specific lipid species known to be related to pathological events, and (2) identification of new prognostic or diagnostic biomarkers able to discriminate with higher specificity and sensitivity the healthy phenotype from the pathological ones. The strength of lipidomic is to identify single species that stand for significant changes and offers broad-spectrum information on the inherently dynamic process. In this process, metabolites and, thus, their concentrations are continuously exposed to synthesis or degradation. By clustering metabolites that are simultaneously interested in changes, it is possible to identify pathways and cell functions involved in the studied stimulus or dysfunction [6]. Among chronic inflammatory and lipids-related diseases, cystic fibrosis (CF) is a significant and well-characterized fatal illness. CF is a pulmonary disease caused by different mutations in the gene for the chloride/carbonate channel CFTR. These mutations are responsible for dysregulation in the electrolytic equilibrium within the protective mucus of respiratory airways, leading to lung chronic inflammation and infections, together with pancreatic insufficiency and multiple organs dysfunction [7]. Pharmacological treatment aimed at CFTR function recovery failed in the clinical practice, and CF has no effective cure at present [8]. Lipid alterations in CF patients have been extensively reported. In particular, abnormalities in blood fatty acid (FA) composition have been described, showing a high level of saturated (SFA) and monounsaturated (MUFA) together with low levels of omega-3 and omega-6 polyunsaturated fatty acids (PUFA) in respect to a healthy control [9,10]. Alteration in the human CF plasma lipid profile comprises a modification in the levels of phospholipids and lysophospholipids (e.g., PC and LPC), cholesterol, cholesterol esters, and hypertriglyceridemia [11,12,13]. In addition, peripheral cholesterol accumulation was evidenced in the respiratory airways [14]. The cause of lipids altered homeostasis in CF is still debated, and it has been attributed to enhanced lipid synthesis that can derive from intestinal malabsorption [12], as well as from peripheral and systemic inflammation [15]. At a cellular level, it was demonstrated that CF cells exhibit increased lipid synthesis, possibly due to altered proteostasis [16,17], which can be counteracted by the sphingolipid synthesis inhibitor myriocin [18,19].

In this manuscript, we present a novel lipidomics approach aimed at identifying those lipid species that unequivocally sign CF pathophysiology. We evaluated the lipid tract of the CF broncho epithelial cell line, being the airways of the first body district involved in chronic inflammation and infection in these patients. Our data strengthen and specify previous reports, demonstrating that CF broncho epithelial cells exhibit a significant increase of all lipid species analyzed in comparison to the normal broncho epithelial cell line. Most importantly, we unequivocally indicate the ether-glycerophospholipids, cholesterol esters, and glycosylated sphingolipids as classes of molecules accurately representative of CF and not well-characterized yet as pathological markers. Our findings open a new bursting and crucial research field for the development of innovative CF therapeutic approaches.

## 2. Materials and Methods

### 2.1. Reagent and Chemicals

Lipids standard were purchased from Avanti Polar Lipids (Alabaster, AL, USA). The chemicals acetonitrile, 2-propanol, methanol, chloroform, formic acid, ammonium acetate, and ammonium formate were purchased by Sigma-Aldrich (St. Louis, MO, USA). All aqueous solutions were prepared using purified water at a Milli-Q grade (Burlington, MA, USA).

### 2.2. Cell Culture

Human bronchial epithelial cell line (IB3), derived from a CF patient (ΔF508/W1282X) provided by LGC Promochem (Teddington, UK), were grown in LHC-8 medium supplemented with 5% FBS, 1% penicillin/streptomycin at 37 °C, and 5% CO_2_. Healthy (H) human lung bronchial epithelial cell line (16HBE14O, initially developed by Dieter C. Gruenert) were provided by Luis J. Galietta (Telethon Institute of Genetics and Medicine—TIGEM, Napoli, Italy). Originally HBE primary cells were grown in LHC-8, although in the present study they were cultured as recommended (Merck Millipore SCC150 datasheet) in MEM Earle’s salt supplemented with 5% FBS, 1% penicillin/streptomycin at 37 °C, and 5% CO_2_. For cell lipidomics, 1 × 10^5^ cells/100-mm plate in 5 mL medium were plated, harvested when confluence has reached 90%, washed in PBS, and pelleted.

### 2.3. Lipids Extraction

Lipid extraction was completed by a modified version of the Folch method [18]. Cells (about 1 × 10^6^) were reconstituted in 100 µL of water + 0.1% proteases inhibitor cocktail, and a small aliquot was used for total protein quantification by the Bradford dye-binding method. For lipid extraction, 100 µL of aqueous samples were added with 850 µL of a methanol/chloroform mixture (2:1, *v*/*v*), then sonicated for 30 min. The organic phase was evaporated under a stream of nitrogen. The residues were dissolved in 100 µL of isopropanol/acetonitrile (2:1, *v*/*v*), centrifuged for 10 min at 13,400 RPM, and withdrawn in a glass vial.

For a targeted sphingolipids analysis, after the addition of the methanol/chloroform mixture (2:1 *v*/*v*), samples were incubated overnight in an oscillator bath at 48 °C. Then, to enhance their recovery, alkaline methanolysis was performed by incubation at 37 °C for 2 h with 75 µL of potassium hydroxide 1 M in methanol. After neutralization with 75 µL of acetic acid 1 M in methanol, samples were evaporated. The residues were dissolved in 100 µL of methanol, centrifuged for 10 min at 13,400 RPM, and withdrawn in a glass vial.

### 2.4. LC-MS/MS Untargeted Method

The LC-MS/MS consisted of a Shimadzu UPLC coupled with a Triple TOF 6600 Sciex (Concord, ON, CA) equipped with Turbo Spray IonDrive. All samples were analyzed in duplicate in both positive and negative mode with electrospray ionization. The instrument parameters were: CUR 35, GS1 55, GS2 65, capillary voltage 5.5 kV, and source temperature 350 °C. Spectra were contemporarily acquired by both full-mass scan from 200–1500 m/z (100 ms accumulation time) and data-dependent acquisition from 50–1500 m/z (40 ms accumulation time, top 18 spectra per cycle 0.8 s). Declustering potential was fixed to 50 eV, and the collision energy was 35 eV, with a collision energy spread of 15 eV.

The chromatographic separation on an Acquity BEH C18 column 1.7 μm 2.1 × 50 mm (Waters, Franklin, MA, USA), equipped with a precolumn [20], was achieved using, as mobile phase A, water/acetonitrile (60:40) and, as mobile phase B, 2-propanol/acetonitrile (90:10), both containing 10-mM ammonium acetate and 0.1% of formic acid. The flow rate was 0.4 mL/min, and the column temperature was 45 °C. The elution gradient was set as below: 0–2 min (45% B), 2–12 min (45–97% B), 12–17 min (97% B), 17–17.10 min (97–45% B), and 17.10–21 min (45% B).

Additionally, another chromatographic separation was reached on an Acquity CSH C18 column 1.7 μm 2.1 × 100 mm (Waters, Franklin, MA, USA) equipped with a precolumn by using, as mobile phase A, water/acetonitrile (60:40) and, as mobile phase B, 2-propanol/acetonitrile (90:10), both containing 10-mM ammonium acetate and 0.1% of formic acid. The flow rate was 0.4 mL/min, and the column temperature was 45 °C. The elution gradient (%B) was set as below: 0–2.0 min (40%), 2.0–2.5 min (40–50%), 2.5–12.5 min (50–55%), 12.5–13.0 min (55–70%), 13.0–19.0 min (70–99%), 19.0–24.0 min (99%), and 24.0–24.2 (99–40%) and kept constant until 30 min. Five microliters of clear supernatant were directly injected in the LC-MS/MS.

### 2.5. Lipidomic Data Processing

The correct identification and relative quantification were attained using MS-DIAL (version 4.0) software [21,22,23]. Data raw files (.wiff) were converted into .abf format in order to perform retention time correction, peak alignment, and identification. The latter was achieved by comparison of experimental spectra with those in the LipidBlast library [24], using both accurate mass and MS/MS fragmentation (Table S1) data (total identification score >70%). Prevalent adducts were previously investigated in our experimental conditions, and thus, the identification was restricted only on them. MS and MS/MS tolerance for peak profile were set to 0.01 and 0.05 Da, respectively. Data were then filtered for blank samples signals with a fold change >10. A quality control pooled sample (QC, a mix of all samples in the batch) was prepared and injected several times (every four samples) during the batch analysis to test the instrumental variability. Lipids that presented a coefficient of variation (CV%) ≥30% in the QC were excluded for further investigation [25]. Then, to restrict biological and analytical variances, normalization was completed by correcting the peak intensities (Equation (1)) of each lipid for both (1) the amount of protein in the extract injected (µg) measured by the Bradford method and (2) the variation in the response of QCs dispersed evenly throughout the batch (by the Lowess algorithm). Lipids containing either a high number of unsaturations or odd-chain fatty acids were manually excluded. An overview of the entire lipid metabolism was represented as the summed amounts, after normalization, of the individual lipids per subclass (an example is shown in Equation (2)).

(1) $${\mathrm{Amount}}_{X}=\frac{\mathrm{Peak}\;\mathrm{intensity}\;\times \;\mathrm{after}\;\mathrm{normalization}}{\mu g\;\mathrm{protein}\;\mathrm{injected}}$$

(2) $${\mathrm{Amount}}_{\mathrm{Cer}}{=\mathrm{Amount}}_{\mathrm{Cer}1\;}{+\mathrm{Amount}}_{\mathrm{Cer}2}{+\mathrm{Amount}}_{\mathrm{Cer}\;n}$$

### 2.6. Statistical and Data Analysis

As a first approach to evidence differences in lipid metabolisms between healthy and CF, the different classes (sum of the concentrations of the species) were compared by *t*-test with GraphPad Prism 7.0 (GraphPad Software, Inc, La Jolla, CA, USA). Then, for biomarker discovery, data tables with the lipids identified under both healthy and pathological conditions were formatted as .csv files and uploaded to the MetaboAnalyst server (version 4.0) [26,27]. Data were checked for integrity, filtered by interquartile range, log-transformed (generalized logarithmic transformation), and auto-scaled. If multiple isomeric lipid species were detected, the sum of their abundances would be further considered. This operation is driven by the fact that the exact position and stereochemistry of the unsaturations could not be deduced from this kind of experiment. The comparison between CF and healthy cells was performed by both univariate and multivariate methods. The volcano plot showed the statistical significance and the fold change of each lipid identified by selecting only those with a *p*-value < 0.05 (corrected for false discovery rate) and a fold change (FC) >2. Partial least squares discriminant analysis (PLS-DA) was performed in order to increase the group separation and investigate the variables with a Variance Importance in Projection (VIP) score >1. These features could be considered as a potential biomarker of CF [28]. The quality of the PLS-DA models was assessed by cross-validation: R^2^ and Q^2^ (i.e., cross-validated R^2^) should be >0.8 in order to avoid overfitting or unreliable estimations [29]. The potential lipids biomarkers were finally determined, combining uni- and multivariate analysis by the combination of the VIP score in the PLS-DA model together with corrected *p*-value and fold change both derived from the Volcano plot. Specifically, it was taken into consideration the products of the VIP score (>1), −log_10_ *p*-value (>1.3), and |log_2_FC| (>1), here named as the impact factor (IF; Equation (3)). Enrichment analysis was performed, on normalized data from MetaboAnalyst, using LION/web by the ranking mode, with a one-tailed Welch 2-sample *t*-test as the local statistics [6]. Changes in lipid patterns between CF and healthy phenotypes were connected to the main branches of LION ontology and, especially, lipid function, cellular component, and physical-chemical properties. The chi-square or binomial tests were used to compare observed with expected data distributions.

## 3. Results

### 3.1. Pre-Analytical Optimization

Folch extraction followed by alkaline methanolysis is the gold-standard for sphingolipids quantification [30,31]. This specific extraction protocol warrants a higher extraction rate of sphingolipids species by suppressing the interferences of preeminent phospholipids [32,33]. Hereby, as expected, it was confirmed that the samples treated with alkaline methanolysis displayed a higher intensity of sphingolipids (Figure S1). Curiously, the procedure used for total lipid analysis yielded a higher number of sphingolipids species correctly identified (84 vs. 104, considering the main subclasses: ceramides, hexosylceramides, and sphingomyelins). The sphingolipids profile, measured as fold changes between the two cell lines, fairly differed when using the two extraction protocols (Figure S1). Taking these results altogether, we decided to avoid the methanolysis in the untargeted lipidomics approach, limiting this specific treatment to the target sphingolipids analysis.

### 3.2. Optimization of the Analytical Conditions for Lipidomics Analysis

Using a mixture of 14 chemically pure lipids (differential ion mobility system suitability kit, synthetic lipid mix, Avanti Polar, Alabaster, AL, USA) covering all the subclasses, two distinct mobile-phase modifiers, and two different columns were tested. Ammonium acetate and an Acquity CSH column gave the maximum peak intensities (Tables S2 and S3) and the best lipidome coverage (Figure S2). CSH column was verified in +34% in lipids identified correctly (995 vs. 741, Figure S2). This was probably related to a better separation of different lipid classes. The number of IDA experiments in a cycle-time was also taken into consideration: using the configuration with 20 spectra/cycle, not surprisingly, the number of total spectra acquired was about two-fold in respect to the top 10 (Figure S3).

### 3.3. Performances of the Untargeted Lipidomics Analysis

MS-DIAL performances were evaluated by running standard samples containing a mixture of chemically pure lipids with a concentration of 1 µg/mL (10 ng injected): 10/14 (65%) lipids were identified correctly matching for both MS and MS/MS data, 3/14 (21%) were identified only by accurate mass, and 1/14 (7%) was not recognized at all (Table S4). The normalization method is critical to balance variations and eliminate experimental or biological biases. Internal standard-based normalization is the gold standard for targeted analysis of metabolites, but for untargeted analysis, it has been demonstrated that the method is out-performed by other approaches. The use of a few selected internal standards is not reasonable for the untargeted analysis of complex biological mixtures, since lipids, also comprising in the same class, displayed different chemical structures (e.g., fatty acid chains) and chromatographic behaviors. The choice of internal standards normalization was for the above reasons avoided.

**Figure 1:**
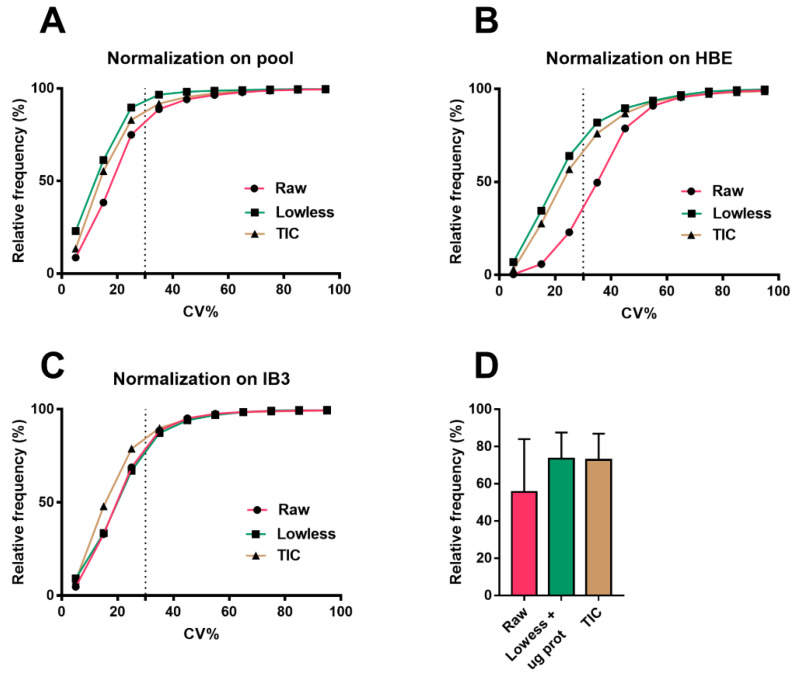
Cumulative frequency distribution of the coefficient of variations (CVs) (%) in (**A**) pool samples, (**B**) healthy HBE, and (**C**) cystic fibrosis (CF) cell extracts obtained for the precision evaluation of different normalization protocols comprehensive of results from both polarities. The dotted line indicates the separation between features within 30% of the CV, which is intended as the maximum permitted for the validation. The graphs showed the better performance of Lowess coupled to µg proteins as the normalization technique, reaching (**A**) 89%, (**B**) 64%, and (**C**) 68% in acceptable features (with a CV% inferior to 30%). (**D**) Graphs show the mean ± SD of the percentage of acceptable features (with a CV% inferior to 30%) between the different normalization techniques.

An alternative approach to reduce the analytical and biological variabilities could be the use of the total ion count (TIC) [34,35]. The TIC was tested in our experiment (ochre curve in Figure 1) and gave satisfactory results with both cell lines, but we noted a limited linearity range in dependence on the amount of proteins in each sample (data not shown).

QC sample (see Methods) was used to calibrate the symmetric biases using weighted scatterplot smoothing (Lowess algorithm on MS-DIAL) for analytical signal correction [21,36,37]. The choice of normalization should be executed with the aim of decreasing variation not only in QC but also in experimental groups [38,39]. Therefore, we lessen the biological variability by normalizing data on the total protein content of the sample. Lowess coupled with biological normalization is presented as a single curve (green) in Figure 1. The latter showed the same performance of TIC, with about 70%–90% acceptable features (CV% < 30%) and, thus, was finally selected for our purpose. These normalization techniques were compared to the raw, not normalized data (red curve in Figure 1). In this limited context, specifically in the comparison between two phenotypes, the different normalization methods demonstrated minimal experimental variations among them, and so we proposed to choose the Lowess coupled with biological normalization (green in Figure 1). Lack of normalization significantly affected the results on the HBE cell line (Figure 1B).

The intra-batch variability, which is the coefficient of variation (CV%) of the QC sample dispersed throughout the batch, was about 16%.

### 3.4. Untargeted Lipidomics of Cystic Fibrosis

MS-DIAL recorded, considering data from both polarities and after blank filtration, n.13375 mass spectra in the whole set of samples, of which, n. 7787 (58%) presented the MS/MS data. The software revealed n.1863-annotated lipids (MS^2^-matched, 14%), and, after a manual validation, the final number was restricted to n.1159 (8.4%), which were grouped in the different classes and subclasses (Figure S4).

**Figure 2:**
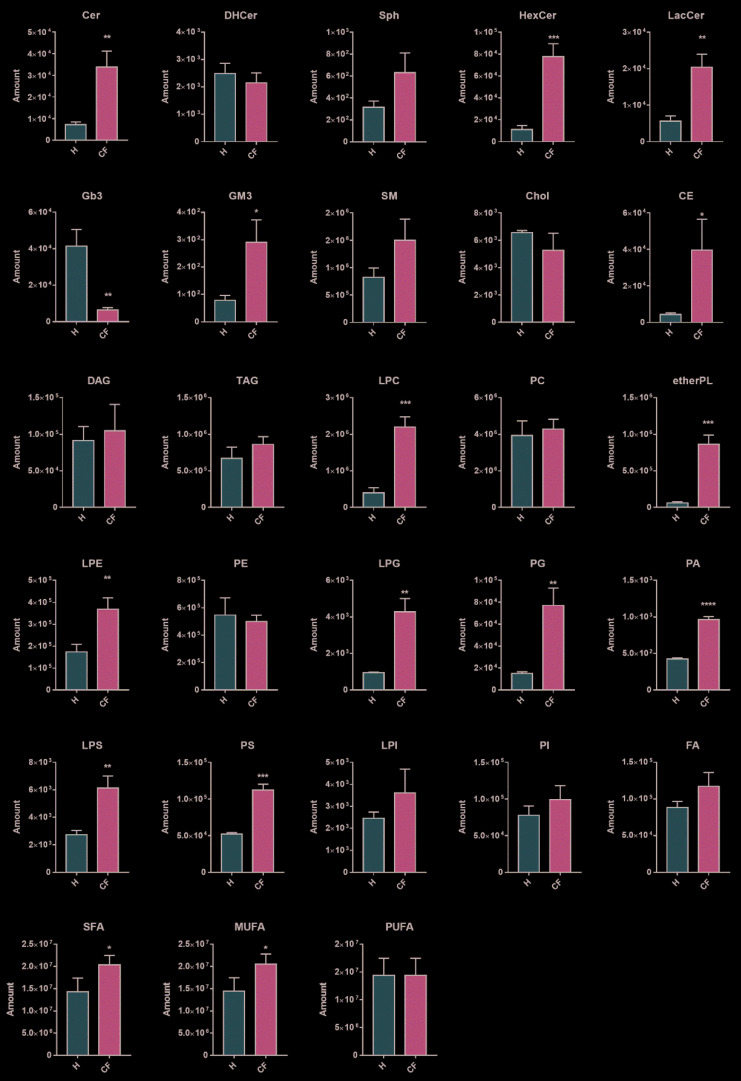
Lipid content comparison between healthy epithelial (H, *n* = 3 independent biological replicates) vs. cystic fibrosis (CF, *n* = 3 independent biological replicates) cells. Graphs represent the lipid amount (Amount, mean ± SD), which indicates the sum of the metabolites intensities within a class after normalization (see Equation (1)). Two-tailed unpaired *t*-tests were performed in each lipid class to establish a statistical difference (* *p* ≤ 0.05; ** *p* ≤ 0.01; *** *p* ≤ 0.001; **** *p* ≤ 0.0001).

The distribution profile of lipid classes in healthy (H, HBE) and cystic fibrosis cells (CF, IB3) was achieved by summing all the normalized intensities of the lipids identified within the single classes (an example is shown in Equation (2)). As expected, in CF, we found a significant general accumulation of all lipid species, in particular ceramides, hexosylceramides, lactosylceramides, GM3, and cholesterol esters (Figure 2). In addition, ether-linked phospholipids (etherPL) were found to be highly modulated by the disease. Specifically, ether-linked phosphatidylcholine (fold change CF/H: 14.56) are the most abundant class recognized in our cell model, followed by ether-linked phosphatidylethanolamine (fold change CF/H: 4.75). No statistical differences were found in the concentrations of free fatty acids, dihydroceramides, sphingomyelins, phosphatidylcholines, phosphatidylinositols, sphingosine, free cholesterol, acylglycerols, cardiolipins (data not shown), and acylcarnitines (data not shown).

**Figure 3:**
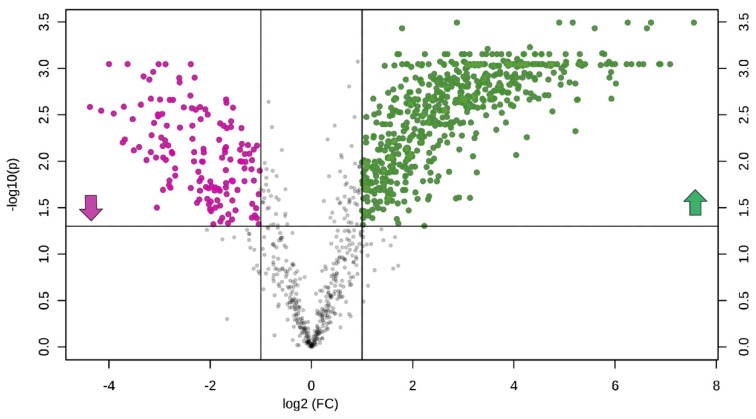
The volcano plot is a combination of fold change and *t*-tests: *X*-axis is log_2_(fold change, FC), and *Y*-axis is −log_10_ (adjusted for false discovery rate). Dots indicate features that presented both a FC >2 and *p*-value < 0.05. Lipids in pink and green are reduced (n.118) and augmented (n.514) in CF vs. H, respectively.

Univariate and multivariate statistical approaches were employed in order to select relevant lipids for cellular phenotype discrimination. Volcano plot analysis selected n. 632 lipids (81.3% elevated and 18.7% reduced in CF vs. healthy), which contemporarily presented a fold change > 2 and a corrected *p*-value < 0.05 (Figure 3).

**Figure 4:**
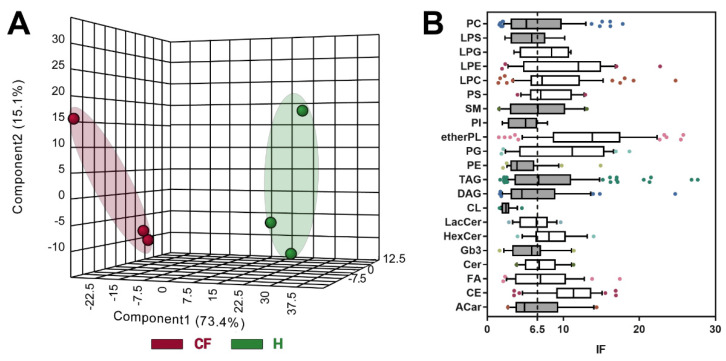
(**A**) Partial least squares discriminant analysis (PLS-DA) chemometric analysis. (**B**) Box and whiskers plots (line at median, and box stretched from the 25–75th percentiles; whiskers indicated the 10–90th, whereas outliers were plotted as single points) of the discriminant lipids (*n* = 624) subdivided for lipid classes and evaluated by their IF scores (see Equation (3)). Grey boxes designated lipid classes that displayed an IF< cut-off (visualized as a dotted line and calculated as the lower confidence limit of the median of the features considered).

Chemometric analysis by supervised PLS-DA (Figure 4A) was then used to maximize the separation between groups and to determine important features of CF by VIP value >1, which were n. 709. Since PLS-DA tends to overfit data, the model was validated [40] by the Leave-one-out cross-validation method, displaying an R2 and Q2 of 0.96 and 0.94, respectively.

Biomarker selection was finally performed combining the data obtained with the different scores from the Volcano plot and PLS-DA. The uni- and multivariate analyses were combined, by restricting features to n.624, in order to increase the discrimination power between the two phenotypes. To achieve this goal, for each identified feature, the IF score (data not shown) was calculated using the VIP score, *p*-value, and FC (see Equation (3)). From the n. 624 lipids, the top 100 discriminant lipids, which distinguished the pathological phenotype of CF from healthy bronchial cells significantly, are listed in Table S5.

(3) $${\mathrm{IF}=|\log}_{2}\mathrm{FC}|\cdot {(-\log}_{10}p\;\mathrm{value})\cdot \mathrm{VIP}\;\mathrm{score}$$

The high presence of lipids bringing an ether-linked acyl chain is shown within this group. In order to have an overview of the main alterations of the lipid CF phenotype, we added a further discriminating analysis that increases the screening of the feature and focuses the attention only on the most significant changes between CF vs. healthy. We proposed the use of the median IF for each lipid class (Table S6), which was graphed as box and whiskers plot (Figure 4B). All the classes were then compared with an arbitrary cut-off (6.5), that we chose to be the lower confidence limit of the median calculated from all the features (n.624). The classes represented by white boxes demonstrated an IF median superior to the cut-off, and therefore, were considered as the most significantly modulated: etherPL, cholesterol esters, and sphingolipids (especially hexosyl- and lactosylceramides).

**Figure 5:**
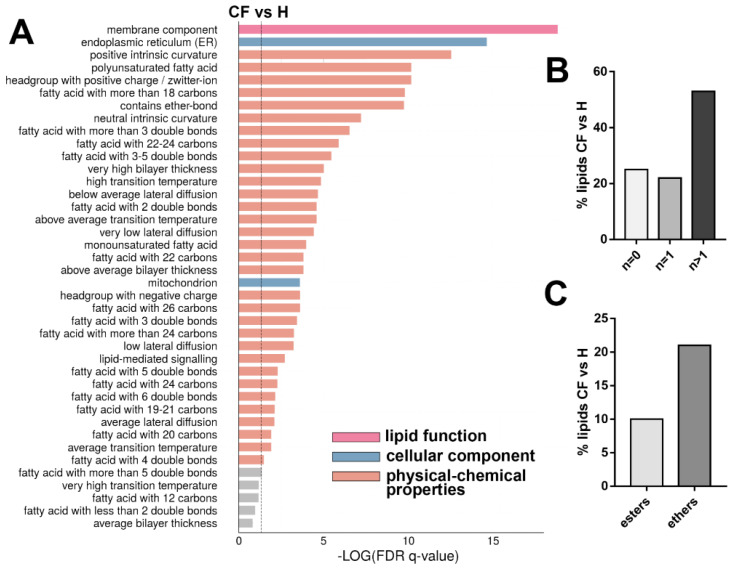
**(A)** Enrichment analysis (top 40) of CF vs. H phenotypes. The dotted line indicates the cut-off value of significant enrichments (*q* < 0.05). Bar length is related with the enrichment (−log q-values corrected for false discovery rate, FDR), whereas colors are dependent to the type of the enrichment: lipid function, cellular component, and physical-chemical properties. (**B**) Distribution of the acyl chain unsaturation from all lipid fraction (%) in CF vs. H discriminant lipid group (top 100). (**C**) Distribution of the ester and ether linkages in phospholipids (%) in CF vs. H in the discriminant lipid group (top 100). In (**B**) the chi-square test and in (**C**) binomial test, revealed a *p*-value < 0.05.

Future validations on the identified biomarkers are highly suggested, possibly on patient-derived primary cells, since this preliminary study analyzed an immortalized cell line. Altered lipid composition, showed in Figure 2, was reflected in different lipid ontologies, indicating lipid function, cellular component, and chemical and physical properties (Figure 5A). The enrichment analysis showed a highly significant modification in the lipids implicated in cell membrane compositions (lipid function ontology). When looking at lipid components, the endoplasmic reticulum and mitochondria compartments are significantly modified in CF vs. healthy cells. Finally, these lipid alterations are also reflected in modifications on chemical and biophysical properties: specifically, affecting chain lengths, saturation, and ether-bound composition of glycerol- and sphingolipids. We noted a quantitative increase in the levels of saturated and monounsaturated fatty acids (SFAs and MUFAs) in CF vs. healthy, whereas the polyunsaturated (PUFAs) species resulted unchanged (Figure 2). Otherwise, in the subgroup of the top 100 discriminant lipids, we observed a prevalence % of PUFAs (Figure 5B). In the same way, we noted a prevalence % of ether-PL over ester-bound phospholipids (Figure 5C).

## 4. Discussion

In this study, we investigated the unusual lipid composition in CF epithelial bronchial cells using an untargeted LC-MS/MS approach. Before each experiment, to ensure that our study produced clinically valid results, we felt the need to carry out a comprehensive optimization study of each step of the method used. We tested two different modified Folch extraction protocols, and we chose the one with the higher number of species identified. We have also highlighted that the use of longer column (10 vs. 5 cm) and with peculiar silica charged surface allows improving the separation between phosphor- and sphingolipids compared to inert silica (BEH). Furthermore, we highlighted the strength of a conservative data-dependent approach in untargeted lipidomics to uncover pathophysiological mechanisms implicated in a disease and, in particular, in CF. It took dedicated time and attention to find the most suitable method for normalizing data before statistical processing, highlighting how this step is particularly critical in biological samples. We propose the use of an innovative statistical index (impact factor) able to combine data from different tests, augmenting the robustness of the discovery results and the consequent biological implications.

CFTR misfunction in CF is associated with altered lipid homeostasis, consisting in inflammatory ceramide accumulation in the lung, sterol accumulation in the airways, hepatic steatosis, and plasma dyslipidemia [11,12,17,41,42,43].

**Figure 6:**
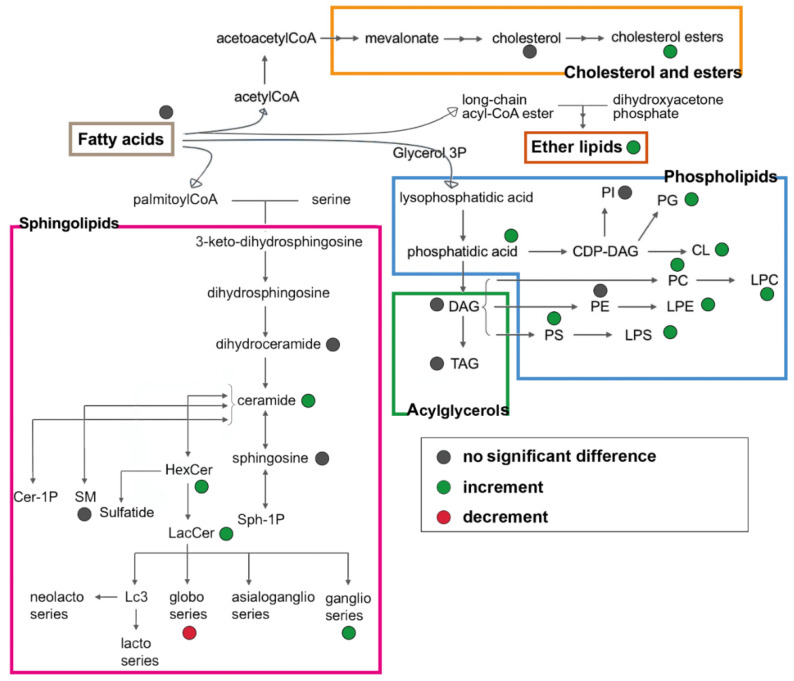
Overview of the lipid biosynthetic and metabolic pathways. Colored dots represented the lipid changes in CF bronchial epithelial cells.

In this preliminary study, we aimed to validate the untargeted lipidomics by the application on the IB3 immortalized CF cell line. Secondly, we accrue to confirm the described lipid alterations and identify the potentially related signatures of the disease (in Figure 6). We strongly feel that our findings on CF lipidomic deregulation need additional confirmation, possibly using patient-derived primary cells or lung biopsies. These data do not attempt to give conclusive findings on the lipid profile in CF but rather to show the importance of a reliable, large-scale analytical method to shed light on biological mechanisms. Despite the use of a single cell line, our results confirm many of the literature findings and disclose interesting topics that deserve further investigations.

The first observation is that, in CF epithelial cells, ceramide and glycosylated sphingolipids accumulate (Figure 2), such as hexosylceramides, lactosylceramides, and GM3 (monosialogangliosides). Ceramides are implicated in inflammation [44,45], and their accumulation, in CF cells, was previously demonstrated by us and others [15,46,47,48]. Studies from our group already demonstrated, through in vivo and in vitro CF models, the therapeutic role of reducing ceramide synthesis [46,49].

Besides, a reduced pool of luminal surface sphingosine, derived from apical membrane ceramide hydrolysis, has been previously reported in CF airways from Grassmé et al. [48], but when looking at the total bulk of cellular sphingosine, we did not observe any significant reduction.

Notably, in this study, using the untargeted lipidomics approach, we observed a significant increase in hexosylceramides (Figure 2). Although poorly characterized up to now, the accumulation of hexosylceramides was demonstrated to exacerbate the inflammation [50,51]; moreover, its synthesis was increased in damaged tissues [52]. In addition, hexosylceramides, which may link either glucose or galactose to ceramide, and lactosylceramides have been associated with oxidative stress and promotion of the inflammatory pathway [53]. The gangliosides (see GM3 in Figure 2) at the membrane were significantly increased. However, the high-ranked discrimination score (IF) found in hexosylceramides (Figure 4B) was not paralleled by polyglycosylated species such as GM3. We speculate that CF cells can enhance ceramide glycosylation to reduce its accrual and related inflammatory stress. The process of glycosylation is also enhanced in tumor metabolism [54]. This hypothesis opens new questions on the physiologic role of this lipid metabolism, and it requires further investigations at the aim of identifying innovative biomarkers in inflammatory diseases and potential therapeutic targets.

Furthermore, we observed an increase in lysophospholipids (Figure 2), specifically in LPC, which was shown to increase during chronic inflammation [55,56]. Mainly, it was found to be incremented in CF bronco epithelial cells, and, for that reason, it can be considered as a possible marker for the chronic inflammatory status [57].

We measured a significant increase in storage lipids, such as cholesterol esters (Figure 2). As previously mentioned, this might be related to the inflammatory status associated with lipid accumulation and to altered lipid intracellular traffic. These data are in-line with reported evidence of cholesterol accumulation in CF bronchi [14,16,58] and, most importantly, with increased concentration of cholesterol esters in pediatric CF patients with bronchoalveolar lavage fluid vs. control subjects [59,60]. Mutated CFTR cells may display an altered lipid synthesis, along with lipid-defective storage, mobilization, and structural usage. These biochemical alterations could sustain chronic inflammation and an inadequate response to infection [19,61].

To note, we also observed a significant increase in etherPL (Figure 2). This finding was also supported by the increased presence of ether-linked fatty acids in lipids extracted from CF bronchoalveolar lavage [56]. Ether lipids, characterized by an ether bond between glycerol and the fatty acid in the *sn-1* position, are essential membrane regulators of fluidity and fusion. Our results suggest that CFTR alteration and disturbance of the membrane composition are somehow linked [62,63]. The increase in ether-link-bearing lipids may be a response to enhanced membrane stability [64]. In addition, it was suggested that ether lipids are involved in regulating cell differentiation, cellular signaling, and reducing oxidative stress by acting as endogenous antioxidants [65].

High levels of SFAs and MUFAs, along with low levels of omega-3 and omega-6 PUFAs, have been reported in CF plasma [9,10,66]. In our CF cell model, we confirmed the quantitative increase of SFAs and MUFAs as compared to control cells (Figure 2). Looking at the top 100 discriminant lipids, however, PUFA species are more represented (Figure 5B) with respect to healthy cells. The observed lipid modifications could impact the biophysical properties of cell membranes, mainly modulating the membrane stability and membrane protein’s function [67,68]. Considering that, in the literature, plasma levels of PUFAs in CF were found to be reduced [9,10,66], however, the link between CF pathophysiology and this abnormality remains unclear [69].

## 5. Conclusions

In this study, we developed an untargeted high-throughput lipidomics workflow and applied it to the study of the unusual lipid composition in a CF epithelial bronchial cell line. We propose the innovative use of the impact factor statistical index to augment the robustness of the discovery, along with the biological and clinical significance. The tested model of the CF bronchial cell line (IB3) displayed a pervasive alteration in the lipid metabolism that, in turn, modified the lipid storage, cell membrane composition, and proinflammatory lipids. Future studies, possibly on patients’ primary cell lines, are required to elucidate our experimental findings further and uncover the pathophysiological mechanisms implicated in CF.

## Supplementary Materials

The following are available online at https://www.mdpi.com/2073-4409/9/5/1197/s1, Figure S1. Sphingolipidomics: comparison between the dedicated extraction of sphingolipid with alkaline methanolysis and total lipid extraction, Figure S2. Comparison between the number of lipids evidenced using two different LC analytical columns, Figure S3. Total number of MS/MS spectra acquired using different data-dependent settings, Figure S4. Distribution of the lipids recognized by lipidomics analysis on the whole set of samples divided by sub-class, Table S1. Lipids identification according to MS/MS fragmentation, Table S2. Performance comparison between mobile phases buffer, Table S3. Performance comparison between analytical columns, Table S4. MS-DIAL performances in the lipid identification, Table S5. Top-100 lipids selected as potential biomarkers associated with CF phenotype, Table S6. Descriptive statistic of the discriminant features divided for lipid classes.

## Abbreviations

H	healthy phenotype
CF	cystic fibrosis
Cer	ceramides
DHCer	dihydroceramides
HexCer	glucosylceramides
LacCer	lactosylceramides
GM3	gangliosides
Gb3	globotriaosylceramide
SM	sphingomyelins
Chol	free cholesterol
CE	cholesterol esters
LPE	lysophosphatidyletanolamines
PC	phosphatidylcholines
PG	phosphatidylglicerols
PI	phosphatidylinositoles
LPI	lysophosphatidylinositoles
PG	phosphatidylglicerols
LPG	lysophosphatidylglicerols
FA	free fatty acids
ACar	acylcarnitines
CL	cardiolipins
etherPL	ether-linked phospholipids
SFA	saturated fatty acids
MUFA	monounsaturated fatty acids
PUFA	polyunsaturated fatty acids
