# Supplementary material for: An Innovative Lipidomic Workflow to Investigate the Lipid Profile in a Cystic Fibrosis Cell Line
Source: Cells. 2020 May 12;9(5):1197. doi: 10.3390/cells9051197 (PMC7291020; doi:10.3390/cells9051197)

Supplementary materials.
An innovative lipidomic workflow to investigate the lipid profile in a Cystic Fibrosis cell line

Michele Dei Cas[a,b], Aida Zulueta[b], Alessandra Mingione[b,c], Anna Caretti[b], Riccardo Ghidoni[b,c], Paola Signorelli[b], Rita Paroni[a]

[a] Laboratory of Clinical Biochemistry and Mass Spectrometry, Department of Health Sciences, Università degli Studi di Milano, 20142, Milan, Italy.
[b] Laboratory of Biochemistry and Molecular Biology, Department of Health Sciences, Università degli Studi di Milano, 20142, Milan, Italy.
[c] Aldo Ravelli Center for Neurotechnology and Experimental Brain Therapeutics, Department of Health Sciences, Università degli Studi di Milano, 20142, Milan, Italy.

Correspondence: Dr Michele Dei Cas, Laboratory of Clinical Biochemistry and Mass Spectrometry, Department of Health Sciences, Università degli Studi di Milano, via A. di Rudini' 8, 20142, Milan, Italy. tel. +39-0250323272/4, email: michele.deicas@unimi.it

Index of contents:

Table S1. Lipids identification according to MS/MS fragmentation. **Table S2.** Performance comparison between mobile phases buffer. **Table S3.** Performance comparison between analytical columns. **Table S4.** MS-DIAL performances in the lipid identification. **Table S5.** Top-100 lipids selected as potential biomarkers associated with CF phenotype. **Table S6.** Descriptive statistic of the discriminant features divided for lipid classes. **Figure S1.** Sphingolipidomics: comparison between the dedicated extraction of sphingolipid with alkaline methanolysis and total lipid extraction. **Figure S2.** Comparison between the number of lipids evidenced using two different LC analytical columns. **Figure S3.** Total number of MS/MS spectra acquired using different data-dependent settings. **Figure S4.** Distribution of the lipids recognized by lipidomics analysis on the whole set of samples divided by sub-class.

Table S1. MS-DIAL achieved lipids identification according to well recognized MS/MS fragmentation pattern as principal adducts found in our experimental conditions.

**Lipid subclasses**	**Adducts**	**MS/MS fragments (Da)**	**Identification**
Ceramides	M+H+	PI 264.26 or 282.27	sphingosine d18:1
Dihydroceramides	M+H+	PI 266.26 or 284.28	dihydrosphingosine d18:0
Sphingomyelins	M+H+	PI 184.07	phosphocholine head group
Neutral glycosphingolipids (HexCer, LacCer, Gb3)	M+H+	PI 264.26 or 282.27	sphingosine d18:1
Acidic glycosphingolipids (GM3, aGM1..)	M+H+	PI 520.5 or 548.5 or 605.5 or 632.5 …	Loss of water by ceramide residues
Phosphatidylcholines	M+H+	PI 184.07	phosphocholine head group
Phosphatidyletanolamines	M+H+	NL 141.01	phosphoethanolamine head group
Cholesterol esters	$M+NH_4+$	PI 369.35	free cholesterol $[M-H_2O+H+]$
Free cholesterol	$M-H_2O+H+$	PI 147.1 or 161.1	
Acylcarnitines	M+	PI 85.0	CH2CH=CHCOOH cation
Cardiolipins	$M+NH_4+$	PI DAG moieties	
Triacylglicerols	$M+NH_4+$		
with FA 16:0		NL 273.2	FA 16:0 + NH_3
with FA 18:0		NL 301.2	FA 18:0 + NH_3
with FA 18:1		NL 299.2	FA 18:1 + NH_3
with FA 18:2		NL 297.2	FA 18:2 + NH_3
with FA 18:3		NL 295.2	FA 18:3 + NH_3
with FA 20:0		NL 329.2	FA 20:0 + NH_3
with FA 22:0		NL 357.2	FA 22:0 + NH_3
with FA 22:1		NL 355.2	FA 22:1 + NH_3
with FA 24:0		NL 385.2	FA 24:0 + NH_3
with FA 24:1		NL 383.2	FA 24:1 + NH_3
Phosphatidylinositoles	M-H-	PI 241.0	Rearrangement of inositol head group
Phosphatidylserine	M-H-	NL 87.0	Serine head group
Phosphatidylglycerols	M-H-	PI 171.0	Glycerols head group
Phospatidic acid	M-H-	PI 152.9	Polar head

NL, neutral loss; PI, product ions

Table S2. Performance comparison between buffers selection (10 mM ammonium acetate vs. 10 mM ammonium formate) in HPLC mobile phases.

A mixture of different lipids (10 ng injected for each lipid, except 25 ng injected for PI) was used to monitor shift in retention times and peak intensities. In the runs with the ammonium acetate, as a buffer, it was assessed a fold-change in the measured lipids of 1.5 (mean) in respect to ammonium formate. Phosphatidylinositol was the only one which displayed a higher intensity with the formate buffer.

**Analytes**	**m/z**	**R_T acetate**	**R_T formate**	**Fold Change in peak height** $_{\text{acetate vs formate}}$
Cholesterol	369.3515	14.06	13.96	**1.05**
SM 18:1	729.5905	14.59	14.55	**1.32**
Cer 18:1	564.5350	15.47	15.48	**1.34**
LPC 18:1	522.3554	3.36	3.19	**1.52**
CE 19:0	689.6207	18.26	18.33	**3.59**
DAG 28:2	526.4466	13.7	13.35	**2.11**
CL 56:6	1233.7917	16.16	16.25	**1.66**
PC 28:2	674.4755	7.66	7.43	**1.27**
TAG 54:3	902.8171	18.9	18.98	**1.15**
PS 28:2	676.41841	6.11	6.00	**1.37**
PE 28:2	632.4285	8.25	7.99	**1.21**
PI 28:2	768.4657	6.32	6.21	**0.74**
PG 28:2	680.4497	6.51	6.36	**1.34**
PA 28:2	606.4129	7.43	7.25	**1.21**

Table S3. Performance comparison between analytical columns (Acquity CSH 1.7 µm 2.1x100 mm *vs* Acquity BEH 1.7 µm 2.1x50 mm) using appropriate LC conditions[1] and the same MS methods. A mixture of different lipids (10 ng injected for each lipid, except 25 ng injected for PI) was used to monitor shift in retention times and peak intensities in ESI+. Phosphatidylinositol and phosphatidic acid were the only which displayed a higher intensity using the BEH.

**Analytes**	**m/z**	**RT CSH**	**RT BEH**	**Fold Change in peak height** $_{\text{CSH vs BEH}}$
Cholesterol	369.3515	14.14	6.73	**1.03**
SM 18:1	729.5905	14.63	7.38	**2.28**
Cer 18:1	564.5350	15.50	8.08	**3.01**
LPC 18:1	522.3554	3.47	1.52	**1.83**
CE 19:0	689.6207	15.90	8.24	**1.57**
DAG 28:2	526.4466	13.85	6.41	**1.34**
CL 56:6	1233.7917	16.16	9.13	**3.91**
PC 28:2	674.4755	7.83	4.82	**1.27**
TAG 54:3	902.8171	18.90	11.52	**7.76**
PS 28:2	676.41841	6.18	4.15	**0.90**
PE 28:2	632.4285	8.42	5.06	**0.92**
PI 28:2	768.4657	6.32	4.08	**0.10**
PG 28:2	680.4497	6.57	4.38	**1.00**
PA 28:2	606.4129	7.44	4.69	**0.26**

[1] BEH and CSH conditions were reported in the main text

Table S4. MS-DIAL performances in the lipid identification.
MS1-matched lipids which were recognized only by their accurate mass, MS2-matched taking into consideration their accurate mass and their MS/MS fragmentation.

**Analytes**	**m/z**	**MS/MS**	**RT**	**Identification**
Cholesterol	369.3515	-	14.06	**MS1**
SM 18:1	729.5905	184.07	14.59	**MS2**
Cer 18:1	564.5350	264.27	15.47	**MS2**
LPC 18:1	522.3554	184.07	3.36	**MS2**
CE 19:0	689.6207	-	18.26	**nd**
DAG 28:2	526.4466	-	13.7	**MS1**
CL 56:6	1233.7917	-	16.16	**nd**
PC 28:2	674.4755	184.07	7.66	**MS2**
TAG 54:3	902.8171	603.53	18.9	**MS2**
PS 28:2	676.4184	491.40	6.11	**MS2**
PE 28:2	632.4285	491.40	8.25	**MS2**
PI 28:2	768.4657	491.40	6.32	**MS2**
PG 28:2	680.4497	491.40	6.51	**MS2**
PA 28:2	606.4129	152.9	7.43	**MS2**

nd, not determined

Table S5. Top-100 lipids selected as potential biomarkers associated with CF phenotype, in decrescent order according to impact factor.

**Analytes**	**m/z**	**class**	**VIP**	**$\log_2$ FC[1]**	**$\log_{10}$ p[2]**	**IF[3]**
TAG 44:0e[4]	754.7312	TAG	1.16	6.71	3.55	27.67
TAG 46:1e	780.7445	TAG	1.16	6.63	3.49	26.85
PC 40:0e	832.7193	PC	1.16	6.25	3.55	25.78
PC 30:2e	688.5293	PC	1.15	7.09	3.11	25.46
LPC 12:0e	426.2983	LPC	1.16	6.87	3.12	24.76
DAG 28:0e	516.4947	DAG	1.15	6.89	3.11	24.71
PC 34:6e	736.5298	PC	1.15	6.56	3.11	23.57
PC 30:1e	690.5464	PC	1.15	6.47	3.11	23.27
LPE 28:0	622.4823	LPE	1.16	6.32	3.11	22.73
PC 42:1e	858.7342	PC	1.16	5.60	3.49	22.67
PC 34:5e	738.5464	PC	1.15	6.23	3.11	22.33
PC 36:6e	764.5612	PC	1.16	6.00	3.12	21.62
TAG 46:0e	782.9629	TAG	1.16	5.79	3.21	21.53
PC 42:2e	856.7151	PC	1.16	5.75	3.21	21.40
PC 30:3e	686.5139	PC	1.16	5.94	3.11	21.35
TAG 44:1e	752.7129	TAG	1.16	5.88	3.14	21.34
PC 44:2e	884.7467	PC	1.16	5.16	3.55	21.29
PC 36:0e	776.6593	PC	1.16	5.83	3.11	20.97
TAG 46:2e	778.7284	TAG	1.15	5.92	3.02	20.57
TAG 52:6e	854.7584	TAG	1.15	5.89	2.97	20.07
TAG 48:3e	804.7456	TAG	1.15	6.02	2.88	19.87
TAG 48:2e	806.7506	TAG	1.15	5.56	3.09	19.82
PC 46:7e	902.6991	PC	1.16	5.30	3.21	19.72
TAG 40:0e	698.6684	TAG	1.15	5.41	3.11	19.44
LPC 12:0	440.2757	LPC	1.16	5.30	3.14	19.23
TAG 42:0e	726.6987	TAG	1.16	5.32	3.12	19.22
PC 36:1e	774.6405	PC	1.15	5.34	3.09	19.05
PG 34:2[5]	747.5203	PG	1.16	5.02	3.21	18.69
TAG 48:1e	808.7694	TAG	1.15	5.19	3.11	18.67
TAG 50:4e	830.7632	TAG	1.14	5.93	2.72	18.36
PC 38:0e	804.6816	PC	1.16	5.03	3.14	18.26
LPC 26:1	634.4817	LPC	1.15	5.03	3.11	18.07
PC 26:0	650.4757	PC	1.15	5.00	3.09	17.82
LPC 28:2	660.4924	LPC	1.16	4.70	3.21	17.47
FA 26:2	391.3578	FA	1.15	4.94	3.06	17.43
PC 44:4e	880.7177	PC	1.15	4.85	3.11	17.40
PC 34:0e	748.6222	PC	1.15	4.99	3.02	17.35
PC 34:4e	740.5577	PC	1.15	5.21	2.88	17.22
TAG 54:0e	894.8869	TAG	1.15	4.78	3.11	17.14
PE 34:6e	694.4787	PC	1.15	4.75	3.11	17.07
TAG 54:1e	892.8713	TAG	1.15	4.75	3.11	17.02
TAG 50:3e	832.7747	TAG	1.15	5.10	2.90	16.99
LPE 26:0	594.4512	LPE	1.16	4.71	3.12	16.98

Analytes	m/z	class	VIP	$\log_2$ FC[1]	$\log_{10}$ p[2]	IF[3]
CE 26:1	780.7583	CE	1.16	4.55	3.21	16.91
PG 32:1e	705.5136	PG	1.16	4.55	3.21	16.90
CE 26:2	778.7456	CE	1.16	4.70	3.11	16.88
LPE 24:0	566.4167	LPE	1.15	4.93	2.97	16.81
LPE 28:1	620.4666	LPE	1.15	4.79	3.02	16.63
PE 30:1e	648.4949	PC	1.15	4.65	3.09	16.57
PC 46:6e	904.7168	PC	1.16	4.42	3.21	16.45
PG 38:5	795.5108	PG	1.16	4.32	3.29	16.45
LPC 26:3	630.4470	LPC	1.16	4.42	3.21	16.44
LPE 22:3	532.3414	LPE	1.15	4.54	3.11	16.28
TAG 50:6e	826.7304	TAG	1.14	5.26	2.70	16.21
PC 26:5	640.3903	PC	1.15	4.93	2.87	16.19
PC 28:1	676.4897	PC	1.16	4.49	3.11	16.15
TAG 52:5e	856.7768	TAG	1.14	5.25	2.70	16.13
PE 32:4e	670.4830	PC	1.15	4.48	3.10	16.03
LPE 12:0	398.2313	LPE	1.16	4.40	3.12	15.86
PG 32:1	719.48608	PG	1.15	4.68	2.93	15.77
PE 32:5e	668.4664	PC	1.15	4.37	3.11	15.68
PG 36:2	773.53088	PG	1.16	4.20	3.21	15.62
CE 26:6	770.6807	CE	1.15	4.50	3.00	15.53
PG 32:0	721.4992	PG	1.15	4.28	3.11	15.36
PG 42:8	845.5235	PG	1.15	4.26	3.11	15.29
TAG 42:1e	624.6833	TAG	1.15	4.25	3.10	15.19
PC 46:1	928.7697	PC	1.15	4.20	3.11	15.10
LPE 22:2	534.3531	LPE	1.16	4.14	3.14	15.02
CE 24:1	752.7251	CE	1.16	4.07	3.17	14.92
PE 30:3e	644.4586	PC	1.15	4.32	3.00	14.91
PE 28:0	636.4586	PC	1.15	4.42	2.93	14.90
CE 26:0	782.7768	CE	1.15	4.18	3.09	14.89
PC 36:2e	772.6211	PC	1.15	4.14	3.11	14.88
DAG 52:5	856.7641	DAG	1.14	4.69	2.76	14.79
PC 46:2	926.7436	PC	1.16	4.11	3.11	14.79
LPC 26:1	634.4782	LPC	1.15	4.30	2.98	14.72
PC 34:1e	746.6063	PC	1.15	4.12	3.09	14.67
TAG 58:1e	948.9314	TAG	1.15	4.21	3.02	14.61
LPC 28:0	664.5289	LPC	1.14	4.61	2.76	14.56
CE 24:2	750.7127	CE	1.15	4.05	3.11	14.53
PC 48:7e	930.7340	PC	1.16	3.90	3.21	14.51
ACar 18:0[6]	428.3742	ACar	1.16	-4.00	3.11	14.38
LPE 22:1	536.3720	LPE	1.15	4.06	3.07	14.38
PC 32:0e	720.5927	PC	1.15	4.01	3.11	14.37
TAG 52:0e	866.8562	TAG	1.15	3.99	3.11	14.31
CE 34:6	882.8071	CE	1.16	3.89	3.14	14.12
LPE 24:6	554.3260	LPE	1.15	3.92	3.11	14.09
PC 44:5e	878.7006	PC	1.15	3.93	3.10	14.07
PG 38:7	791.4871	PG	1.16	3.86	3.14	14.02

Analytes	m/z	class	VIP	log_2 FC[1]	log_{10} p[2]	IF[3]
HexCer d18:1, 24:1	810.6830	HexCer	1.15	4.12	2.96	13.99
PC 34:3e	742.5757	PC	1.16	3.75	3.21	13.97
LPC 18:0e	510.3906	LPC	1.15	4.02	3.01	13.93
DAG 30:1e	542.5146	DAGe	1.14	4.49	2.72	13.91
CE 32:6	854.7753	CE	1.15	3.88	3.11	13.90
LPE 14:0	426.2549	LPE	1.15	3.86	3.11	13.88
TAG 52:7e	852.7459	TAG	1.13	4.76	2.56	13.84
LPC 28:3	658.4807	LPC	1.16	3.79	3.15	13.84
PC 36:5e	766.5722	PC	1.16	4.06	2.97	13.83
FA 28:1	421.4017	FA	1.15	4.34	2.78	13.75
TAG 50:5e	828.7439	TAG	1.12	5.22	2.35	13.72

[1] Fold-change was always referred as CF/H cells (cystic fibrosis /healthy phenotype)
[2] p value was corrected for false discovery rate
[3] see equation 3
[4] e indicates the presence of ether- instead of ester-linkage
[5] underlined numbers indicates lipid revealed under ESI-
[6] ACar 18:0 was the only specie in the top-100 lipids which displayed a decrement in the CF phenotype

Table S6. Descriptive statistic of the discriminant features (n=624) divided for lipid classes. Others indicate a miscellanea of various lipids (n.21) in different classes with a neglectable importance.

class	n.	min	25th	median	75th	max	Mean	SD	SEM	CV%
ACar	10	2.71	3.82	4.91	9.22	14.38	6.34	3.78	1.19	59.58%
CE	36	3.51	9.15	11.34	13.59	16.91	10.93	3.48	0.58	31.85%
Cer	12	3.83	5.04	6.83	8.88	11.34	7.01	2.40	0.69	34.19%
CL	15	1.61	1.98	2.44	2.80	4.55	2.58	0.75	0.19	29.07%
DAG	37	1.65	3.18	4.55	8.85	24.71	6.33	4.82	0.79	76.10%
FA	26	2.09	3.73	6.99	10.23	17.43	7.44	3.96	0.78	53.21%
Gb3	15	1.61	3.38	5.86	6.97	11.37	5.68	3.01	0.78	52.96%
HexCer	12	3.98	6.42	8.11	10.21	13.99	8.43	2.63	0.76	31.22%
LacCer	14	2.84	4.31	6.87	7.83	9.65	6.22	2.00	0.54	32.23%
LPC	56	1.44	5.77	7.19	12.09	24.76	8.90	4.75	0.64	53.39%
LPE	28	1.94	4.80	11.99	14.86	22.73	10.57	5.66	1.07	53.51%
LPG	5	3.50	4.37	8.46	10.64	10.99	7.69	3.24	1.45	42.04%
LPS	9	2.32	3.16	5.85	7.53	10.17	5.73	2.59	0.86	45.12%
PC	69	1.58	3.18	5.14	9.67	17.82	6.69	4.15	0.50	61.92%
etherPL	59	1.51	8.68	13.83	17.40	25.78	13.39	6.31	0.82	47.10%
PE	22	2.05	3.14	3.93	6.07	14.90	5.08	3.01	0.64	59.12%
PG	26	1.86	4.27	11.17	15.31	18.69	10.36	5.23	1.03	50.41%
PI	5	1.97	2.79	5.02	6.47	7.91	4.71	2.19	0.98	46.53%
PS	14	3.98	5.72	7.02	11.03	12.89	7.86	2.92	0.78	37.13%
SM	10	1.53	3.08	6.55	10.14	13.08	6.71	3.84	1.22	57.29%
TAG	123	1.68	3.84	6.48	11.85	27.67	8.62	5.76	0.52	66.12%
Others	21	-	-	-	-	-	-	-	-	-

Figure S1. Sphingolipidomics: comparison between the dedicated extraction of sphingolipid with alkaline methanolysis (Ext sph) and total lipid extraction (Ext tot).
(**A**) Fold-change (FC) of the main sub-classes of sphingolipids (ceramides, hexosylceramides and sphingomyelins) within the two extraction protocols: the alkaline methanolysis increase the intensity (about 2-fold) of the species due to the removal of the interferences of phospholipids in the extract. Fold-change of the concentration of (**B**) ceramides, (**C**) hexosylceramides and (**D**) sphingomyelins in cystic fibrosis (CF) and healthy phenotypes (H) as the function of the extraction protocols. Taking into account these results, the extraction methods appear to be comparable.

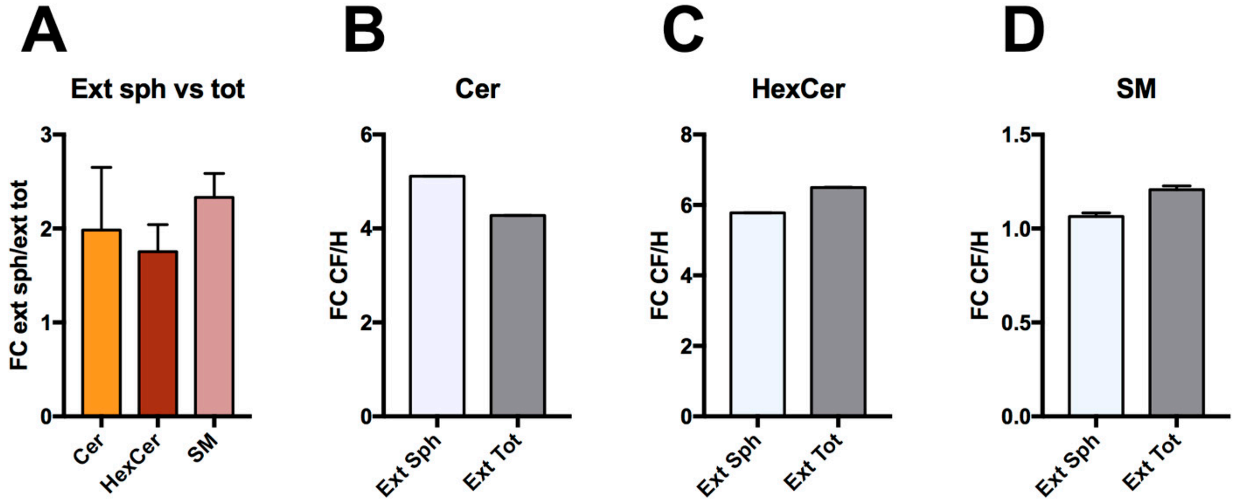

Figure S2. Comparison between the number of lipids evidenced using two different LC analytical columns: BEH (50 *x* 2.1 mm, 1.7 µm) vs CSH (100 *x* 2.1 mm, 1.7 µm). CSH showed better performance, with respect to the other configuration, with a +34% in the lipidome coverage.

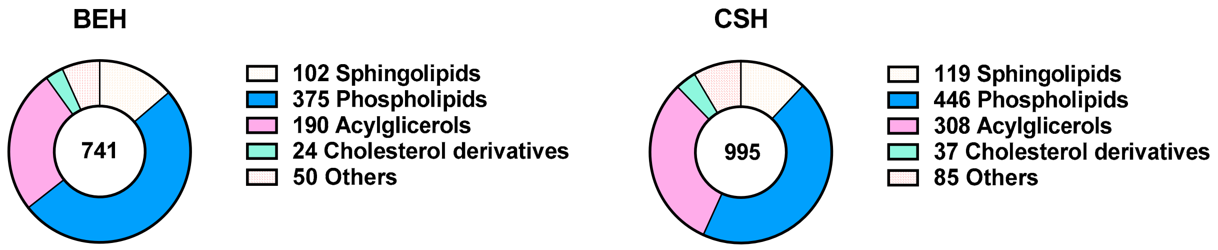

Figure S3. Total number of MS/MS spectra acquired using data-dependent top-10, top-18 and top-20 settings.

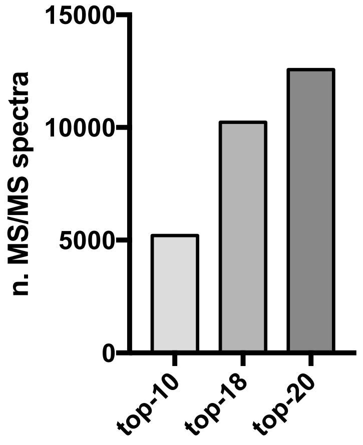

Figure S4. Distribution of the lipids recognized by lipidomics analysis on the whole set of samples divided by sub-class.

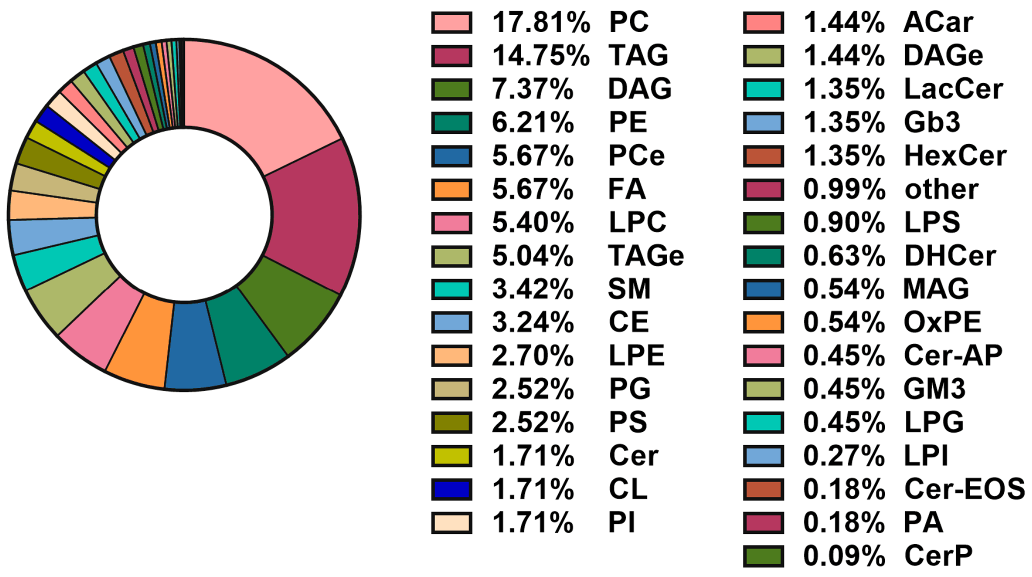

Supplement: Supplementary file 1 [file cells-09-01197-s001.pdf]
